# EMT and stemness: flexible processes tuned by alternative splicing in development and cancer progression

**DOI:** 10.1186/s12943-016-0579-2

**Published:** 2017-01-30

**Authors:** Davide Pradella, Chiara Naro, Claudio Sette, Claudia Ghigna

**Affiliations:** 1 Istituto di Genetica Molecolare – Consiglio Nazionale delle Ricerche, via Abbiategrasso 207, 27100 Pavia, Italy; 2 Dipartimento di Biologia e Biotecnologie, ‘Lazzaro Spallanzani’ - Universita` degli Studi di Pavia, via Ferrata 9, 27100 Pavia, Italy; 3 Department of Biomedicine and Prevention, University of Rome Tor Vergata, 00133 Rome, Italy; 4 Laboratory of Neuroembryology, Fondazione Santa Lucia, 00143 Rome, Italy

**Keywords:** Alternative splicing, EMT, Stem cell differentiation, Cancer stem cells, Tumor progression, RNA binding proteins

## Abstract

Epithelial-to-mesenchymal transition (EMT) is associated with metastasis formation as well as with generation and maintenance of cancer stem cells. In this way, EMT contributes to tumor invasion, heterogeneity and chemoresistance. Morphological and functional changes involved in these processes require robust reprogramming of gene expression, which is only partially accomplished at the transcriptional level. Alternative splicing is another essential layer of gene expression regulation that expands the cell proteome. This step in post-transcriptional regulation of gene expression tightly controls cell identity between epithelial and mesenchymal states and during stem cell differentiation. Importantly, dysregulation of splicing factor function and cancer-specific splicing isoform expression frequently occurs in human tumors, suggesting the importance of alternative splicing regulation for cancer biology.

In this review, we briefly discuss the role of EMT programs in development, stem cell differentiation and cancer progression. Next, we focus on selected examples of key factors involved in EMT and stem cell differentiation that are regulated post-transcriptionally through alternative splicing mechanisms. Lastly, we describe relevant oncogenic splice-variants that directly orchestrate cancer stem cell biology and tumor EMT, which may be envisioned as novel targets for therapeutic intervention.

## Background

Epithelial cells are typically immobile cells, characterized by an apical-basal polarity with cohesive cell-cell junctions connecting adjacent cells in a continuous monolayer [1]. On the contrary, mesenchymal cells exhibit a motile and invasive phenotype by adopting an elongated shape with a front-back polarity [2]. Epithelial-to-mesenchymal transition (EMT) is a developmental program underlying the acquisition of mesenchymal properties by epithelial cells [3]. This process is fundamental during embryogenesis, when regulated migration of restricted population of cells is required for organogenesis [4]. In adult mammals, activation of EMT is mainly exploited in wound healing. However, this process is also reactivated by cancer cells to invade adjacent tissues and to disseminate toward distant organs, representing an essential step during progression of epithelial cancers to more aggressive stages [4]. Furthermore, EMT has also been involved in generation of cancer stem cells (CSCs) [5], the subpopulation of cells identified within leukemias and solid tumors as having self-renewal and expanding capability, thus contributing to tumor growth, metastasis and resistance to conventional therapies [6].

EMT relies on profound changes in gene expression that require multiple layers of regulation, from transcription, to post-transcriptional RNA processing, to translational and post-translational modifications. Although transcriptional regulation by EMT-inducing transcription factors (EMT-TFs), like members of the ZEB, SNAIL and TWIST families, is generally considered the master step in this process, mounting evidence indicates that post-transcriptional events strongly contribute to the fine-tuning of EMT [7]. Notably, post-transcriptional mechanisms of gene expression regulation have recently emerged as important tools exploited by cancer cells to acquire unique features that confer advantages over surrounding cells and sustain tumor malignancy [8]. In this regard, splicing of precursor messenger RNAs (pre-mRNAs) appears particularly suited to fine-tune regulation of gene expression because of its extreme flexibility.

It is clear that alternative splicing (AS) of pre-mRNAs plays an essential role in generating proteome diversity in cancer cells, through the production of splice-variants involved in key oncogenic pathways and resistance to chemotherapeutic drugs [9–11]. The advent of next generation sequencing and the development of highly specific bioinformatics tools have offered the possibility to study AS regulation with increasing detail. Through these approaches, a number of cancer-specific AS isoforms have been identified [12], paving the ground for their application in cancer diagnosis and as targets for selective anti-cancer treatments.

AS regulation modulates several molecular and morphological processes involved in EMT [13, 14]. Since AS is a versatile and powerful mechanism to both establish and maintain fundamental properties of different cell and tissue types [15, 16], it is not surprising that it contributes to promote the plasticity required for the EMT process and for establishing the stem-like properties that typify the more aggressive nature of neoplastic cells.

In this review, we offer a brief overview of EMT programs in development, stem cell biology and cancer progression. Subsequently, we assess the contribution of AS in EMT, describing interesting examples of both splicing factors and target genes, and presenting AS profiles that contribute to the dynamic transitional states between the epithelial and mesenchymal phenotypes in cancer. We also focus on the impact of AS regulation in cellular features that are directly related to the oncogenic potential of CSCs and provide examples of AS variants involved in acquisition and maintenance of stem cell-like features.

## Main text

### Epithelial-mesenchymal transition: a flexible tool for cell plasticity during embryogenesis

EMT was first characterized during embryonic development when a restricted population of epithelial cells differentiate into motile mesenchymal cells in order to form new tissues at specific sites, leading to the three-dimensional organization of developing organs [3, 4].

Differentiation of three embryonic layers during gastrulation of avian and mammalian embryos is the proto-typical example of a developmental program relying on EMT. Epiblastic cells of the primitive streak undergo EMT to move internally and generate the two inner layers of mesoderm and endoderm, while differentiation of the remaining epiblast generates the ectoderm [17]. EMT also promotes migration of neural crest cells from the epithelium near the dorsal midline of the neural tube towards prescribed embryonic regions where they differentiate to give rise to ganglia of the peripheral nervous system and other neural-derived cell types [18]. Interestingly, once their final target destination is reached neural crest cells re-aggregate through a reversible process of mesenchymal-to-epithelial transition (MET), which interrupts cell migration inducing these cells to form novel epithelial tissues [17, 19]. Notably, gastrulation and neural crest migration represent just two of the many examples of EMT/MET processes occurring during embryogenesis, since several rounds of reversible EMT and MET are necessary for proper embryo development [20].

Signals from multiple cues orchestrate the proper execution of EMT/MET cycles during embryogenesis. An example of signaling molecule involved in these programs is provided by WNT, whose signaling pathway promotes EMT and ingression of epiblastic cells from the primitive streak during gastrulation [21]. Furthermore, WNT acts synergically with other regulatory molecules, such as BMP4, for the induction of EMT in the migratory neural crest cells during their delamination from the neural tube [22]. These signal transduction pathways ultimately induce the expression of EMT-TFs [7]. Indeed, both gastrulation and neural crest cell migration require increased expression of SNAIL1 and SNAIL2 (also known as SLUG) [23, 24], which mediate repression of the epithelial adhesion protein E-cadherin, leading to the disruption of adherens junctions (AJ) and acquisition of a mesenchymal migratory morphology. Downregulation of E-cadherin is not sufficient to induce EMT phenotypic changes, and regulation of other adhesion molecules is often required. For example, repression of both E-cadherin and CAD6b coupled with upregulation of less adhesive type II cadherins, such as cadherin 7 and 11, is required during neural crest cell migration [25, 26]. Likewise, another EMT-TF, ZEB1, regulates the E- to N-cadherin switch occurring during the transition from the pre-migratory to the migratory state of the neural crest cells [27], an event necessary for activation of directional migration [28]. Another key step in EMT is the digestion of the extracellular matrix (ECM) of the basal membrane. This process allows the complete detachment of the cells from the original epithelial layer and their migration towards the novel site of destination. Degradation of the ECM is mainly mediated by membrane-bound and/or secreted forms of matrix metalloproteases (MMPs) [29], such as the MMP-2, which also contribute to EMT-driven events during embryogenesis [30].

### Molecular processes involved in EMT

Epithelial integrity is ensured by specialized cell-cell junctions organized through the assembly of cell surface protein complexes: adherens junctions (AJ), tight junctions (TJ) and desmosomes (DS) [31]. TJ are mainly responsible for the sealing of the epithelial layer and acquisition of apico-basal polarity [32]. Transmembrane proteins, such as MARVEL-domain proteins, occludins, claudins and junctional adhesion molecules (JAMs) mediate cell-cell adhesion, whereas cytosolic proteins (mainly zona occludens family members, ZO1/2/3) stabilize the junction by binding cytoskeleton components and providing the docking sites for polarity proteins (PAR3, PAR6, PALS1 and PATJ), signaling components (aPKC, CDC42, RAC and RHOA) and their regulators (RHOGEFs and RHOGAPs) [33].

AJ, similarly to DS, display cadherin clusters as core components [34]. Cadherins are transmembrane proteins that allow cell-cell adhesion among adjacent cells [35]. Both TJ and AJ are able to interact with the actomyosin machinery and this association plays critical functions for cytoskeleton organization and cell-shape remodelling [36]. Mechanistically, the link between the junction and actin or microtubule filaments is provided by catenins (β-catenin, p120 and α-catenin) [37]. Cadherin-catenin clusters facilitate the recruitment of cytoskeletal regulators and polarity proteins to the junctional complex [34, 38].

**Fig. 1:**
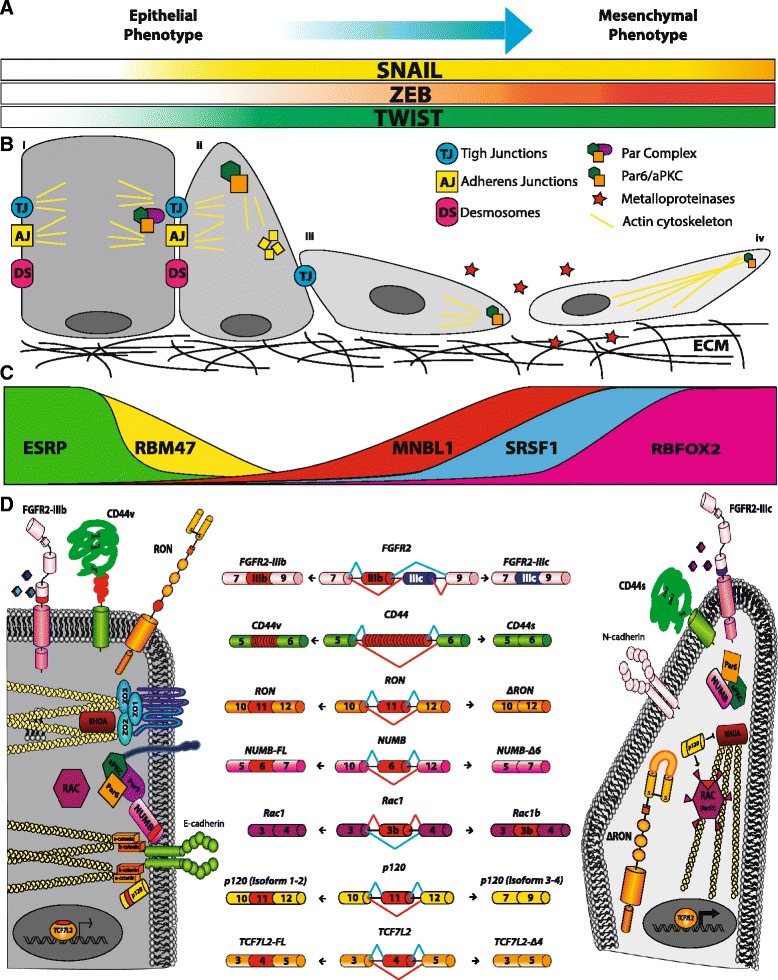
Significant alternative splicing changes occurring during EMT. **a** Key transcription factors upregulated during EMT; gradient color represents their expression increase from epithelial to mesenchymal phenotype. **b** Schematic representation of EMT progression. From left to right: (*i*) polarized epithelial cell with strong cell-cell junctions. Par complex and actin filaments localize to the junctions; (*ii*) epithelial cell with residual junctions starts to re-organize its cytoskeleton and change its morphology. E-cadherin disappears from cell membrane (*small yellow square*). The Par complex is disassembled and PAR6/aPKC move to the apical cell surface; (*iii*) the epithelial cell loses its epithelial features and begins to acquire an elongated and spindle-like morphology, while PAR6/aPKC, with other polarity complexes (not shown), allow the establishment of a front-rear polarity. Metalloproteases are secreted in order to degrade the ECM; (*iv*) a motile mesenchymal cell is able to invade the surrounding tissues. **c** Expression gradients of key splicing factors regulated during EMT. **d** *Center*. AS of genes involved in different EMT programs, including migration and invasion (*FGFR2*, *RON* and *CD44*), polarity and cytoskeleton organization (*NUMB*, *RAC* and *p120*) and transcription regulation (*TCFL2)*. Alternative exons are represented in red, mutually exclusive exon in blue. *Left*. Scheme of epithelial-specific AS variants. Alternative exons and the encoded amino acids are indicated in red. *Right*. Mesenchymal-specific isoforms are also shown. Differences in functional properties of epithelial versus mesenchymal isoforms are highlighted: *FGFR2* exons IIIb and IIIc confer different ligand binding specificity; ΔRON and Rac1b are constitutively active cytoplasmic isoforms; inclusion of exon 6 in *NUMB* allows it to interact with Par complex and E-cadherin; p120 isoforms 1-2 localize to AJ, whereas p120 isofoms 3-4 localize with the activate RAC and repress RHOA signaling thus promoting re-organization of the actin cytoskeleton; skipping of exon 4 in *TCFL2* generates the more active transcriptional factor TCFL2-Δ4

The prevailing models for EMT regulation propose that a sequential series of events are required for an epithelial cell to acquire mesenchymal features [7] (Fig. 1a, b). During the first step, TJ are disassembled by complete abrogation of occluding and claudin expression [39]. Together with the loss of the transmembrane backbone of the junction, the cytoplasmatic components (ZO1/2/3) diffuse away from cell-cell contacts [40]. In addition, loss of E-cadherin is another fundamental event in EMT [41]. Specifically, E-cadherin is degraded by proteolytic cleavage or through endocytosis from the plasma membrane [42, 43], whereas its expression is repressed (directly or indirectly) by EMT-TFs [44]. As result of E-cadherin disappearance from the cell membrane, catenins are free to move in the nucleus where they act as transcriptional regulators of specific mesenchymal genes [45].

Disappearance of apical-basal polarity is another strictly coordinated event in EMT, which involves both transcriptional repression [46] and re-localization of key cytoskeletal components to the leading edge of the cell. For instance, regulation of Par (PAR3/PAR6/aPKC) and Scribble (Scribble/LGL/DLG) complexes, which specify apical membrane identity, as well as of the Crumbs (PALS1/PATJ/Crumbs) complex, which specifies basal membrane identity, promotes a shift toward a front-rear polarity [47]. Simultaneously, lamellipodia, filopodia and invadopodia are formed by actin cytoskeleton remodeling mediated by the CDC42 and RAC signaling pathways [48]. Globally, these changes shift cell morphology toward a motile and invasive phenotype. Finally, expression of MMPs [29], which degrade the ECM, together with the appearance of mesenchymal markers (N-cadherin, Vimentin, Fibronectin, α5-Integrin) complete the transition to a motile cell that is able to colonize distant tissues [45] (Fig. 1a, b).

The acquisition of mesenchymal properties during EMT occurs progressively along an axis, wherein fully epithelial and mesenchymal cells represent the extreme edges [7]. This plastic and dynamic process comprises several intermediate states, including hybrid phenotypes in which cells concomitantly express epithelial and mesenchymal features [1, 49]. Importantly, cells carrying such hybrid epithelial/mesenchymal phenotype (referred as hybrid E/M) not only exert fundamental roles in embryogenesis, but also during cancer progression [50, 51].

### Role of EMT in cancer

During malignant progression of epithelial cancers, tumor cells acquire an invasive and motile phenotype in order to invade adjacent tissues and disseminate toward distant organs. This metastasis formation process is responsible for approximately 90% of cancer mortality [52]. Notably, metastasis is a highly inefficient process. Indeed, it has been estimated that, from 10,000 tumor cells that enter the circulation, only one is able to develop a macroscopic metastasis [53]. Since tumor epithelial cells have cohesive cell-cell junctions that inhibit their movements, the transition toward a mesenchymal phenotype through activation of EMT has been proposed as a key step for tumor dissemination and cancer progression [3]. Although it was initially believed to occur in advance stages of cancer progression, supported by the positive correlation between tumor size and metastatic potential [54], it is now recognized that tumor dissemination and micrometastases can be found in early stages of the disease [55]. Accordingly, epithelial cells undergoing EMT have been found in pre-neoplastic lesions of pancreatic tissues [56]. As in the course of embryonic development, tumor EMT is a reversible process, and regain of epithelial features through MET can also occur at the final metastatic site [57].

Various cues in the tumor microenvironment are implicated in establishing an intricate network of interactions that activate the EMT/MET programs [58]. Cancer cells are associated with a large array of stromal cells, including fibroblasts, myoblasts, macrophages and lymphocytes, but also with endothelial cells and pericytes recruited to the tumor vasculature [59]. Paracrine and juxtacrine signals in such microenvironment include growth factors and cytokines [60]. In addition, oxidative stress, hypoxia and morphogenic (NOTCH and WNT) signaling pathways increase expression of EMT-TFs. The combined action of these signals, together with the nature of the ECM components, induces cancer cells to adopt molecular and morphological features of either epithelial or mesenchymal identity [61]. EMT in cancer progression follows the same pattern described for physiological EMT programs, with disruption of cell-cell adhesion, loss of polarity and cytoskeleton reorganization, release of mesenchymal-specific MMPs (MMP-1, MMP-2, MMP-9, MMP-12 and MMP-13) and degradation of the ECM that allows invasion of the original tissue and dissemination [62–64]. Notably, high levels of MMPs in the tumor microenvironment affect both stromal and cancer cells. Stromal cells are induced to produce additional MMPs (MMP-7 and MMP-14), thus increasing the degradation of the ECM and promoting tumor invasion [65]. Moreover, MMPs can mediate the proteolytic cleavage of E-cadherin, generating extra-cellular E-cadherin fragments that increase motility [66]. Importantly, expression of different types of MMPs is associated with worse prognosis in several cancers, including ovarian [67], breast [68], gastric [69] and colorectal cancers [70].

EMT has also been linked to other aspects of cancer biology such as inhibition of cellular senescence [71] and chemoresistance [72, 73]. An interesting example is provided by ZEB1/2. These EMT-TFs are induced by TGF-β and repress the cyclin kinase inhibitors p15^INK4B^, p16^INK4A^ and p21, thus abolishing EGFR-dependent senescence in esophageal squamous cell carcinoma [74]. Similarly, TWIST cooperates with Ras signaling to prevent oncogene-induced cellular senescence through abrogation of p53- and Rb-dependent pathways [75]. Finally, reduced susceptibility to apoptosis during EMT is conferred by the action of EMT-TFs on survival pathways, mainly MEK/ERK and PI3K/AKT [76], and pro-apoptotic and anti-apoptotic genes, such as the Bcl2 family members [77].

Activation of EMT has been associated with chemoresistance in different tumor types. Enrichment of cells expressing mesenchymal markers has been detected in breast, colorectal and non-small lung cancers upon chemotherapeutic treatments [78–80]. In line with these observations, inhibition of EMT-TFs and post-transcriptional regulators of EMT was found to abrogate EMT-induced chemoresistance in breast and pancreatic cancer models [72, 73]. Chemoresistance might result from the combined activation of the many cellular processes involved in EMT and may be related to acquisition of stem-like features by cancer cells. High expression of the EMT-TFs ZEB1 [81], SNAIL1 and SNAIL2 [82] in cancer cells triggers the expression of stemness factors SOX2 [81], BMI1 and OCT4 [6, 81, 82]. Notably, mesenchymal and stemness traits are known to characterize the CSC subpopulation within the tumoral mass, which is responsible for tumor metastasis and resistance to conventional therapy [6]. Thus, EMT might revert the phenotype of terminally differentiated epithelial cells to a more plastic, mesenchymal phenotype that mirrors some properties of pluripotent embryonic cells during organogenesis.

EMT has been shown to be a transient process occurring only in a subset of cells at the invasive front of the primary carcinoma, usually associated with stromal components [83]. Nevertheless, hybrid E/M cells have been found in different tumors, including breast, ovarian and lung cancers [84–86] and in some tumor mouse models [56, 87]. Accordingly, circulating tumor cells (CTCs) with a fully mesenchymal state display lower metastatic potential compared to hybrid E/M cells that underwent a partial EMT [88]. A more heterogeneous expression of mesenchymal and epithelial markers is detected in CTC clusters, which are aggregates of 2-50 tumor cells held together through intercellular adhesions and recruitment of platelets [88]. CTC clusters are also characterized by a high metastatic potential taking advantage of both mesenchymal properties, which sustain cell motility and invasion [51], and epithelial features involved in extravasation and colonization propensity [89]. Notably, it was recently reported that also breast CSCs showing an hybrid E/M state, characterized as CD24− CD44+ ALDH+, displayed the highest invasive ability [90]. These observations strongly suggest that maintenance of a transient epithelial-mesenchymal phenotype reflects an increased cellular plasticity, which allows acquisition and preservation of stemness traits by cancer cells. This hypothesis is also supported by several recent studies showing that, in addition to EMT, the MET pathway can also induce stem-like properties and increase metastatic potential in cancer cells. For instance, downregulation of EMT-TFs in prostate and bladder cancer cells was reported to promote expression of stemness factors and to enhance their growth as spheroids [91], the typical pattern of stem cell growth [92]. Similarly, silencing of PRRX1, a transcription factor that induces EMT, promotes the acquisition of stem cell properties by breast cancer cells, enhancing their self-renewal ability and growth in mammospheres [93].

### Mechanisms of regulation of EMT: the emerging role of alternative splicing

EMT requires a robust reprogramming of gene expression [3]. Several EMT-TFs are activated early during EMT to either repress epithelial-specific genes or induce specific mesenchymal features. Epithelial-specific genes, such as *E-cadherin*, *claudins* and *occludins*, are repressed by SNAIL proteins (SNAIL1 and SNAIL2) [41, 94]. SNAIL1/2 bind to epithelial-specific promoters, recruit several epigenetic regulators such as PRC2 [95], HDAC1/2 [96], LSD1 [97], G9a [98] and SUV39H1 [99], and promote chromatin condensation [100]. Similarly, bHLH transcription factors (TWIST1 and TWIST2) and ZEB proteins (ZEB1 and ZEB2) are able to both repress epithelial genes and stimulate expression of mesenchymal-specific genes, such as *N-cadherin*, *fibronectin* and *matrix metalloproteases* [101–103].

**Fig. 2:**
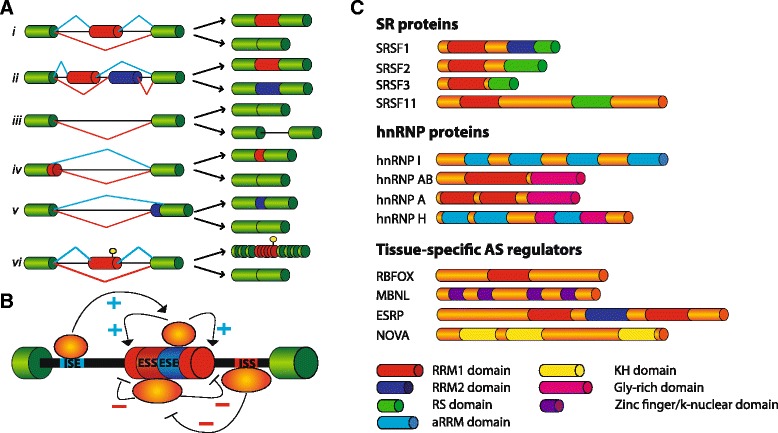
Alternative Splicing regulation. **a** Scheme of the different AS modalities: (*i*) cassette exons; (*ii*) mutually exclusive exons; (*iii*) intron retention; (*iv*) alternative 5′ splice sites; (*v*) alternative 3′ splice sites; (*vi*) inclusion of a poison exon containing a premature stop-codon (*yellow*) leading to mRNA degradation through NMD. Precursor transcripts and final spliced products are shown. **b** AS regulation by combined action of *trans*- and *cis*-acting elements. Intronic and exonic splicing enhancers (ISE and ESE) promote the inclusion (+) of the AS exon (*red*) by providing the binding sites for activators (*orange circles*), whereas intronic and exonic splicing silencers (ISS and ESS) are bound by repressors (*yellow circles*) and promote exon skipping (-). Generally, ESE-bound SR factors stimulate the assembly of the splicesome on the variant exon or counteract the inhibitory activity of hnRNPs bound to ESS elements. On the contrary, hnRNPs interfere with the assembly of spliceosome to the variant exon leading to exon skipping. In addition, hnRNPs by binding ISSs located in the introns flanking a variant exon cause its looping out and skipping, whereas when bound to ESSs they may polymerize along the exon and displace the ESE-bound SR proteins (not shown). **c** Some members of the SR and hnRNP families mentioned in the text are shown with their characteristic domains. SR proteins have a modular structure with one or two RNA recognition motifs (RRM) in the N-terminus able to interact with the pre-mRNA, whereas at C-terminus all members of this family present a domain of variable length rich in serine-arginine dipeptides (RS domain) involved in protein-protein interactions with spliceosomal components. HnRNPs possess one or more RNA-binding domains associated with different “auxiliary” domains that are diverse in sequence and involved in sub-cellular localization or protein-protein interactions. Tissue-specific AS regulators (RBFOX, MBNL, ESRP and NOVA families) are indicated with their own RNA-binding domains

However, the proteomes of mesenchymal compared to epithelial cells show significant differences [104] only partially explainable by functions of EMT-TFs. Mounting evidence suggests that post-transcriptional events, and in particular AS, significantly contribute to this diversity. Splicing occurs during transcription, and in some instances post-transcriptionally, when intronic regions are removed by direct interactions of the splicing machinery (the spliceosome) with short, poorly conserved, *cis*-acting sequence elements at exon–intron boundaries (donor or 5′ and acceptor or 3′ splice sites). This poor level of conservation allows high flexibility in splice site recognition, with frequent competition between sites showing variable strength [105, 106]. By using different combinations of donor and acceptor sites, more than 90% of human genes are able to generate different mRNAs through AS of selected exons and introns (Fig. 2a), thus yielding an estimated number of at least 100,000 different proteins [107]. Regulation of AS is modulated by the action of *cis*-acting elements (non-splice site RNA sequence elements) and *trans*-acting factors on the pre-mRNA. *Cis*-acting elements promote (splicing enhancers) or inhibit (splicing silencers) the usage (or definition) of variable exons by providing binding sites for *trans*-acting splicing regulators. *Cis*-elements can be found alone or clustered in introns (ISE/ISS, intronic splicing enhancer/silencer) as well as inside exons (ESE/ESS, exonic splicing enhancer/seilencer) (Fig. 2b) [105]. Serine-arginine (SR) factors and heterogeneous ribonucleoproteins (hnRNPs) are the two major classes of ubiquitously expressed *trans*-acting splicing factors [105]. SR proteins are highly conserved splicing regulators characterized by the presence of a C-terminus serine rich domain (the RS domain) implicated in protein-protein interactions [108] (Fig. 2c). By binding to splicing enhancers, typically purine rich motifs [109] through their RNA-recognition motifs (RRM), SR proteins usually promote exon recognition stabilizing spliceosomal components at exon–intron boundaries or antagonizing splicing repressor (Fig. 2b). However, SR proteins are also able to stimulate exon skipping suggesting that their activity is influenced by a complex network of interactions with the others RNA binding proteins (RBPs) expressed in specific cell types and/or development stages [110, 111]. Similar to SR proteins, hnRNPs have a modular structure with RNA-binding domains flanked by auxiliary domains with different functions and properties (Fig. 2c). Generally, hnRNPs bind to splicing silencers preventing the association of SR proteins or spliceosome components to alternative exons [112], thus leading to exon skipping (Fig. 2b). While SR proteins and hnRNPs are widely expressed across different tissues and cell types, other splicing factors display a cell-type-specific pattern of expression. To date, the best characterized mammalian tissue-specific AS regulators are NOVA1/2, PTBP2 (also known as nPTB or brPTB), SRRM4 (nSR100) and members of the RBFOX, MBNL, CELF, TIA, ESRP and STAR families (Fig. 2c). For some of these factors, the mode of action during the AS reaction is very peculiar since it depends on the position of their binding sites on pre-mRNA targets. For instance, NOVA1/2 proteins are able to promote exon inclusion when they bind to *cis*-acting elements (YCAY clusters) located in exons or near the 3′ splice site of the intron, while they promote exon skipping if their binding sites are located near the 5′ splice site [113]. The tissue-specific expression pattern of these splicing factors help establish the appropriate spatio-temporal generation of splice variants in many cellular and developmental processes [114, 115]. Since some excellent reviews have recently illustrated the general mechanisms of AS regulation, the reader is referred to them for additional insight [106, 116].

Transcription and AS coordinately control different subsets of genes to generate the molecular and cellular complexity of cell and tissue types [15, 16, 106, 117]. Thus, it is not unexpected that AS also contributes to the dynamic (molecular and morphological) cellular conversion during EMT [118]. In line with this notion, expression of several splicing factors has been reported to be modulated during EMT [119]. Since each of them is able to regulate hundreds of pre-mRNA targets, it is likely that perturbation of their expression levels can simultaneously affect different aspects of EMT progression [7].

### The ESRP splicing factors: key regulators of epithelial identity

A salient example of how EMT can be modulated by expression of specific splicing factors is provided by ESRP1 and ESRP2, two epithelial-restricted splicing regulators [120–122]. ESRP gain- and loss-of-function cells and genome-wide based approaches were used to characterize the ESRP-dependent epithelial splicing signature and its contribution to EMT [122]. These high-throughput approaches uncovered an important role of the ESRP-mediated RNA network in affecting exons of genes involved in RNA splicing, vesicle-mediated transport system, cell polarity, cell junction organization, motility and migration, regulation of small GTPase-mediated signal transduction and actin cytoskeleton [14, 121–123]. In addition, this analysis decoded the RNA map by which ESRP1/2 regulate AS. Indeed, similar to NOVA1/2 and RBFOX2 [113, 124], ESRP proteins display a positional effect and promote or repress exon inclusion depending on the locations of their binding sites (UGG-rich motifs) in RNA targets [14].

One of the best characterized ESRP targets is the *Fibroblast growth factor receptor 2* (*FGFR2*) pre-mRNA. ESRPs control mutually-exclusive regulation of two exons (IIIb and IIIc) encoding a protein domain with critical roles in ligand binding specificity [120]. Splicing of these exons ensures the appropriate expression of FGFR2 isoforms and, as a consequence, the correct FGF/FGFR2 signaling during development. Moreover, altered splicing of exons IIIb and IIIc in *FGFR2* pre-mRNAs was found in primary tumors and metastases and it was associated with tumor plasticity [125]. ESRPs repress exon IIIc and increase inclusion of exon IIIb, leading to production of the epithelial-specific FGFR2-IIIb isoform. On the contrary, downregulation of ESRP proteins promote the inclusion of the mesenchymal-specific exon IIIc and, at the same time, induce molecular and morphological changes associated with EMT progression [120, 122]. In order to properly regulate AS of *FGFR2* pre-mRNAs, ESRPs cooperate with other widely expressed RBPs, including PTBP1 (hnRNP I), hnRNP A1, M, F and H [126–129]. Thus, the net outcome of *FGFR2* AS in any given cell depends on the specific repertoire of splicing factors expressed. These observations suggest that multiple cues could modulate this EMT-related splicing event by affecting expression or post-translational modifications of splicing factors involved in this regulation.

An interesting observation is that, in several cases, ESRP-regulated splice variants exhibit distinct and even opposing functions during EMT. The *p120* pre-mRNA splicing event that generates two variants (p120 isoforms 3 and 4) is able to promote cell-cell adhesion in epithelial cells by increasing p120 binding to E-cadherin in AJ [130]. In contrast, the mesenchymal-specific p120 isoform 1 induces cell migration and invasiveness by inhibiting RHOA-ROCK signaling pathway and stimulating RAC1 activity [131]. Another example of ESRPs target is *NUMB* pre-mRNA, which encodes for a factor involved in maintenance of cell polarity and cell-cell adhesion by binding to Par polarity complex and E-cadherin, respectively [132]. Through its N-terminal phosphotyrosine binding domain (PTB) domain, NUMB binds a conserved NVYY motif in the cytoplasmic portion of E-cadherin. Tyrosine phosphorylation of this motif abolishes NUMB/E-cadherin association, allowing NUMB to interact directly with the Par complex members PAR6 and aPKC [133]. Interestingly, 11 amino acid residues of the PTB domain are encoded by an epithelial-specific exon whose inclusion is controlled by ESRP proteins [122]. Skipping of this exon has been proposed to affect NUMB cellular membrane localization as well as its interaction with E-cadherin, resulting in loss of cell-cell adhesion [122].

ESRPs also regulate cell polarity through AS regulation of *SCRIB* transcripts [121]. SCRIB is a scaffolding protein required for epithelial cell identity and prevents EMT progression by blocking loss of E-cadherin and ZO1 from AJ [134]. In contrast with these roles, SCRIB knockdown has been associated with impaired cell migration and downregulation of mesenchymal markers [135]. The apparent antithetical functions of SCRIB in cell migration and EMT could be partially explained by the ESRP-dependent splicing of *SCRIB* pre-mRNAs, where SCRIB epithelial-isoform is required for AJ stability, whereas the mesenchymal-specific variant is involved in cell motility [136]. Splicing changes of ESRP target exons also affect actin cytoskeleton organization and its regulators. The *ENAH* gene generates an epithelial-specific splice variant, derived from inclusion of a small exon (exon 11A) encoding 21 amino acids in the C-terminal Eva/Vasp homology (EVH2) domain [137]. Downregulation of this variant was linked to tumor invasiveness in vivo [138], whereas a mesenchymal specific isoform lacking exon 6 (ENAH-Δ6) was associated with invasiveness in mesenchymal-like breast tumors [139]. In addition, ENAH interacts with ABI1, another ESRP target gene involved in actin cytoskeleton remodelling and cell-cell adhesion [140]. Remarkably, ESRP-mediated AS of *ABI1* pre-mRNA influences the sequence of the proline region domain important to mediate ABI1 association with several partners, including ENAH [140].

Other ESRP targets include cell membrane proteins such as integrins and receptors (KITLG, MPZL1, ITGA6, CD46, CD44) that are able to sense environmental signals, but also components of signaling pathways involved in EMT (MAP3K7, SOS1 and FYN) [122]. Moreover, ESRPs could act indirectly on expression levels of epithelial transcripts, as they stimulate inclusion of exon 4 of the *TCF7L2* transcription factor, thus promoting an isoform with reduced ability to activate β-catenin-target genes in epithelial cells [141]. Additionally, ESRP-mediated AS of *ITGA6*, *CD46* and *MAP3K7* variant exons causes introduction of premature stop-codons able to induce mRNA degradation through non-sense mediated decay (NMD) [122], a process known as alternative splicing activated NMD (AS-NMD) [142].

Many ESRP-regulated pre-mRNA targets encode proteins that interact with each other (Fig. 1c, d). This observation suggests that ESRPs control a network of epithelial regulators and that AS plays an important role in affecting physical interactions between these factors during activation of EMT programs. Hence, the phenotypic changes reported upon ESRPs knockdown are likely the integrated effects of several AS changes that may act in a coordinated manner. Considering the essential role of ESRPs in coordinating epithelial cell-type-specific AS programs, several groups have investigated how their expression level are regulated. Collectively, it was proposed that downregulation of ESRPs can be induced by transforming growth factor (TGF)-β-induced EMT [143, 144], epigenetic mechanisms [145] and gene mutations [146]. Notably, ESRP1 is among the most downregulated genes in different EMT experimental models [119, 144, 147–149], indicating that its presence may represent an obstacle to acquisition of mesenchymal features. In line with this hypothesis, the EMT-TF ZEB1, which is upregulated in several human cancers [150, 151], directly inhibits ESRP1 expression, thus causing AS changes in the *CD44* gene [120]. *CD44* encodes a cell surface glycoprotein that binds different components of the extra-cellular matrix [152]. Repression of ESRP1 by ZEB1 promotes expression of a mesenchymal CD44 splice variant (*CD44s*) [153]. Importantly, switch from epithelial isoforms (CD44v) to CD44s was proposed to play a role in EMT [154]. Notably, ZEB1 downregulation was associated to a more invasive phenotype in lung cancer [153], suggesting that ZEB1-induced EMT and ESRP1-mediated splicing of *CD44* could contribute to initial transitions of the metastatic progression. On the other hand, increased expression of ESRPs is linked to better survival in colorectal cancer [155], whereas ESRP1 upregulation is proposed as a favorable prognostic factor in pancreatic ductal adenocarcinoma [156]. In addition, during squamous cell carcinogenesis expression levels of ESRPs seem to be very dynamic with their downregulation observed at the invasive front of the tumor and re-expression in lymph node metastases [157]. All these finding suggest that changes in expression levels of ESRP proteins and, as a consequence, the dynamic regulation of AS of their targets, could contribute to EMT plasticity during malignant transformation.

### Other splicing factors involved in EMT/MET processes

In addition to ESRPs, other splicing factors contribute to EMT-associated AS changes [14] (Fig. 1c, d). RBM47, an RBP involved in pre-mRNA splicing, mRNA stability and RNA editing [158, 159], is downregulated during EMT. Notably, RBM47 regulates many splicing cassette exons in the same direction of ESRPs, suggesting a functional combinatorial co-regulation between these splicing factors to promote epithelial splicing patterns [14]. However, some AS events seem to be regulated with opposing effects by ESRPs and RBM47, thus indicating a more complicated pattern of interactions between these proteins during EMT.

If ESRP proteins are fundamental to establish an epithelial-specific splicing program, RBFOX2 and MBNL1 are important contributors of the mesenchymal splicing signatures [160] (Fig. 1c, d). In particular, expression of RBFOX2 is induced during EMT [119], whereas *Rbfox2* transcripts were found more abundant in normal mesenchymal tissues compared to epithelial ones [161]. Notably, RBFOX2 downregulation causes a partial reversion in cell morphology and motility towards an epithelial phenotype [119, 149] and these defects correlate with AS changes in an organizer of actin cytoskeleton (*Cortactin*), a polarity protein (*PAR3*) and a component of the vesicle-mediated transport system (*Dynamin 2*) [149]. Interestingly, some of the RBFOX2 targets (*NUMB* and *MAP3K7*), for which RBFOX2 promotes the production of the mesenchymal-specific isoform, are also regulated by ESRPs in epithelial cells in order to generate their epithelial-specific protein [122]. However, in other cases RBFOX2 was also found to promote epithelial splicing [149]. This scenario is further complicated by the recent observation that RBFOX2 cooperates with Quaking (QKI), an RBP of the STAR (signal transduction and activation of RNA) family [162], in the splicing regulation of common pre-mRNA targets [14].

MNBL1 is another RBP that regulates mesenchymal-specific AS profiles [119]. For instance, MNBL1 cooperates with RBFOX2 in generating mesenchymal isoforms of *ADD3* and *LRRFIP2* genes, whereas a competition between MNBL1 and PTBP1 is present in the case of *PLOD2* and *INF2* pre-mRNAs [161]. Interestingly, MNBL1 is also implicated in other aspects of RNA metabolism [163, 164]. It has been recently reported that MBNL1 promotes the mRNA stability of two genes involved in metastasis suppression (*DBNL* and *TACC1*) and this effect was linked to breast cancer metastatic colonization, a cancer type where MBNL1 expression was associated to metastasis-free survival [165].

Members of the SR family, such as SRSF1 and SRSF3, are also regulated during EMT and play a role in its progression [166, 167] (Fig. 1c, d). SRSF1 (historically known as SF2/ASF) is upregulated in many human tumors and its over-expression increases cell proliferation, delays apoptosis and is sufficient to transform human and mouse mammary epithelial cells in vivo and in vitro [168, 169]. Upregulation of SRSF1 occurs through different mechanisms acting at the transcriptional [170], post-transcriptional [171, 172] and post-translational levels [168, 173]. Notably, in the past our group has contributed to demonstrate that SRSF1 expression levels are dynamically controlled in epithelial and mesenchymal cells through AS-NMD of an intron in the 3′UTR of the *SRSF1* gene. In particular, AS-NMD of *SRSF1* transcripts, which is altered in colon cancer, is controlled through the STAR protein SAM68 [171], a RBP linked to neoplastic transformation and tumor progression [174, 175]. At post-translational level, SRSF1 activity is instead regulated through phosphorylation by kinases that are often upregulated in human cancers [176], such as SRPK1 [166] and NEK2 [177]. Upon phosphorylation, SRSF1 localizes to the nucleus [178] where it modulates AS of several genes involved in motility and invasiveness [10]. Among SRSF1 pre-mRNA targets, splicing of the proto-oncogene *RON* was the first example of an AS event linked to EMT activation [110]*.* RON is a tyrosine kinase receptor that activates a signaling cascade leading to cell dissociation, migration and matrix invasion [179]. Interestingly, the constitutively active ΔRON isoform, generated through AS of a cassette exon of 147 nucleotides, is able to confer increased motility to the cell [180] and it is frequently over-expressed during tumor progression of epithelial cancers [110, 181]. SRSF1 stimulates skipping of exon 11 and promotes the production of ΔRON, which in turn activates the EMT program [110]. Importantly, ΔRON production is also promoted by hnRNP A2/B1, another hnRNP proteins involved in EMT and altered in several cancers [182, 183], whereas it is inhibited by hnRNP A1, which in this way activates the reversal MET program [184]. In parallel, the cancer associated ΔRON splice variant was analyzed as a potential target for the development of new anti-cancer therapeutic strategies. Bifunctional antisense oligonucleotides or small-molecule inhibitors of SRSF1 activity showed a positive effect in correcting *ΔRON* splicing toward an increase exon 11 inclusion [185]. Notably, in addition to preventing the production of the *ΔRON* isoform, inhibitors of SRSF1 activity were also able to affect the invasive phenotype of the cells [185]. Several additional splicing targets of SRSF1 have now been identified by RNA-seq in breast cancer cells [186]. Among them, SRSF1 stimulates the production of the constitutive active variant of the *Rac1* gene (called Rac1b), which is generated from the inclusion of a highly conserved cassette exon [187] and is characterized by an increased Rac GDP/GTP exchange activity [188]. Rac1b, expressed in several tumors [189], affects the EMT process in different ways: by increasing reactive oxygen species (ROS) and subsequently inducing the EMT-TF SNAIL [190]; by upregulating of the mesenchymal marker Vimentin [190]; or bypassing oncogenic induced senescence in lung and colorectal cancer [191, 192]. Interestingly, ESRPs contribute to repression Rac1b expression splicing in epithelial cells emphasizing, once again, the integrated effects of several AS factors to determine the epithelial or mesenchymal identity.

### AS in stem cell differentiation

EMT represents a typical example of cellular plasticity, which promotes differentiation from one phenotype to another during developmental or pathological programs. The cell types displaying the highest extent of plasticity in our body are the stem cells. Thus, it is not surprising that these cells exploit molecular processes that amplify the flexibility and plasticity of their genome, like AS. Indeed, recent evidence has linked AS regulation to stem cell biology and some remarkable examples are reported below.

Stem cells are undifferentiated pluripotent cells, which are distinguished from other cells because of their ability to asymmetrically divide, to either self-renew themselves or to generate cells committed to differentiation towards a specific cellular lineage [193]. AS of specific genes can modulate the balance between self-renewal and differentiation in response to developmental or environmental cues, thus influencing the developmental potential of tissues and organs [194].

In the last decade, several studies based on high-throughput sequencing have uncovered genome-wide AS programs regulated during differentiation of pluripotent embryonic stem cells (ESCs) into different cellular lineages [195–197]. Moreover, widespread splicing variations have been also observed during differentiation of multi- and unipotent stem cells, as occurring during neurogenesis [198], hematopoiesis [199] and myogenesis [200, 201]. Notably, global changes in AS patterns also occur during the in vitro derivation of ESCs from the inner cell mass of blastocysts [202], suggesting that widespread AS reprogramming are not only required during the differentiation of stem cells, but also for the acquisition of their stemness features. This notion is also supported by high-throughput analyses of transcriptome changes during the cell reprogramming [203–205]. These analyses revealed that reprogramming of somatic cells to induced pluripotent stem cells (iPSCs) is accompanied by a progressive reversion of their splicing profile toward one that closely resemble that of pluripotent ESCs [203]. Intriguingly, orthologous genes display evidence of high conservation in the AS patterns activated during ESCs differentiation and iPSCs induction [160, 197, 206], further supporting an important evolutionary role of AS regulation in the biology of stem cells. Splicing changes occurring during iPSCs induction do not just reflect the phenotypic transition taking place, but they play an active role in reprogramming, as demonstrated by the ability of iPSCs specific splice-variants of the *Ccne1* and *Grlh1* to enhance acquisition of stemness by somatic cells [204, 207]. Importantly, the splicing program activated during iPSCs reprogramming is reversible, as iPCSs redifferentiation to somatic cells leads to re-establishment of the original somatic splicing profile [160]. Overall, these observations highlight the pivotal role of AS in the flexible and reversible regulation of gene expression operated by stem cells upon their switch between self-renewal and differentiation.

**Fig. 3:**
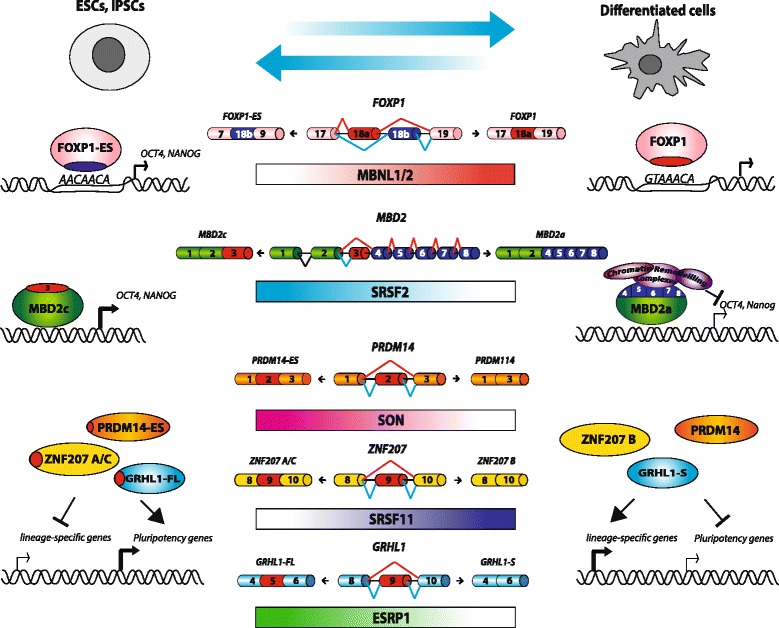
Significant alternative splicing changes occurring during stem cell differentiation. *Center*. Splicing factors and AS of genes involved in somatic cell reprogramming; gradient color represents splicing factor expression increase/decrease from ESCs or iPSCs to differentiated cells. *Left*. Scheme of ESCs or iPSCs-specific AS variants. Alternative exons and the encoded amino acids are indicated in red. *Right*. Differentiated cells-specific isoforms are also shown. Differences in functional properties of pluripotent versus differentiated isoforms are highlighted: *FOXP1* mutually exclusive exons confer different DNA binding properties; *MBD2* AS variants *c* and *a* are both enriched at the promoters of Oct4 and Nanog, but only MBD2a is able to recruit chromatin remodeling complexes to repress pluripotency factors transcription; PRDM14-ES, ZNF207 A/C and GRHL1-FL enhance somatic cells reprogramming, whereas their AS isoforms, lacking the alternative exon, counteract reprogramming

One of the major mechanisms by which AS regulates stem cells biology is the generation of splice-variants of key factors controlling the balance between pluripotency and differentiation (Fig. 3). In this regard, an interesting example is represented by the transcription factor FOXP1. Pluripotent ESCs and iPSCs exclusively express a specific FOXP1 splicing isoform (FOXP1-ES), which includes exon 18b and encodes for a protein isoform having different DNA binding properties with respect to the canonical factor expressed in differentiated somatic cells [197]. Differently from the somatic isoform, FOXP1-ES activates the expression of pluripotency genes, such as *Oct4* and *Nanog*, and its expression is critical for self-renewal and pluripotency of ESCs, as well as for efficient iPSC reprogramming [197] (Fig. 3). Likewise, pluripotent stem cells preferentially express MBD2c, an AS variant of the methyl-CpG binding protein MBD2a that is mainly expressed by differentiated cells [208]. While both proteins are enriched at the promoters of *Oct4* and *Nanog*, only MBD2a is able to interact with repressive chromatin remodelling complexes (Fig. 3). Accordingly, MBD2a overexpression negatively regulates transcription of core pluripotency factors in iPSCs, whereas MBD2c enhances somatic cells reprogramming [208]. Splice variants with different pluripotency capacity have been described also in other key transcriptional regulators of pluripotency, such as OCT4 [209] and NANOG [210], thus further highlighting the importance of AS in expanding the coding capability of transcriptomes in regulating stem cells biology.

AS may also affect proliferation and differentiation of stem cells by regulating steady-state expression levels of specific mRNAs. Indeed, recent advances in next-generation sequencing technologies have revealed that pervasive intron retention coupled to NMD and other nuclear RNA surveillance mechanisms control developmentally-regulated expression of selected gene subsets during differentiation of multi- and uni-potent stem cells, including neurogenic [211] and hematopoietic [212] lineages. For instance, during early phases of neurogenesis, the splicing factor PTBP1 promotes intron retention of 3′-terminal introns within genes encoding for presynaptic proteins. Intron retention targets these transcripts to nuclear degradation and prevents their precocious expression during neuronal development. Then, the progressive decrease of PTBP1 expression during neuronal differentiation allows splicing of regulated introns, thus ensuring the appropriate developmentally controlled expression of target mRNAs [211]. Consistently with the great impact exerted by AS regulation in the control of the balance between pluripotency and differentiation of stem cells, different genome-wide RNA interference (RNAi) screenings identified several RBPs and RNA processing factors as key regulators of self-renewal properties of stem cells [203, 213–215]. In particular, search for critical genes required for the reprogramming of mitotic cells in iPSCs identified splicing factors SON [214] and SRSF11 [215]. Notably, these splicing factors behave as crucial players with opposite functions in the acquisition and maintenance of stemness. While SON enhances somatic cell reprogramming and positively regulates maintenance of stemness, SRSF11 acts as a repressor and negatively regulates the acquisition of the stemness phenotype. Both studies also revealed putative splicing targets involved in somatic cells reprogramming. SON regulates splicing of a critical pluripotency transcriptional regulator PRDM14, thus promoting a long isoform containing exon 2 that enhances reprogramming [214]. SRSF11 promotes exon 9 skipping in ZNF207, leading to a shorter isoform that counteracts reprogramming [215] (Fig. 3).

Bioinformatics analyses of potential regulators of the AS changes observed in stem cells and differentiated cells revealed additional splicing factors as critical regulators of the balance between self-renewal and differentiation [160, 205, 206]. Search for binding motifs enriched within AS events regulated between stem and somatic cells discovered MBNL1 and MBNL2 proteins as possible major regulators [206]. Accordingly, MBNL1 and MBNL2 are scarcely expressed in ES cells and actively repress stem-specific AS variants in differentiated cells [206]. In particular, MBNL1 and MBNL2 inhibit FOXP1-ES splicing [206] and their overexpression impairs iPSCs induction [160, 206]. Similarly to MBNL1/2, also RBFOX2 negatively regulates production of stem cell-specific splice variants and its overexpression inhibits somatic cells reprogramming [160] (Fig. 3). On the other hand, ESRP1 enhances reprogramming of somatic cells into pluripotent cells. Putative binding sites for this protein were found upstream of exons undergoing skipping during somatic cells reprogramming. As an example, ESRP1 promotes splicing of the longest isoform of *Grlh1*, which enhances cell reprogramming [205].

The importance of RBPs and splicing factors for the regulation of stem cell fate is also supported by knock-out mouse models. For example, genetic ablation of *Ptpb1* causes embryonic lethality shortly after implantation [216, 217], while depletion of its paralog *Ptpb2* impairs the embryonic splicing program required for neuronal maturation [218]. Establishment and maintenance of the AS programs accompanying stem cell fate requires the controlled expression of the splicing factors that regulate these programs. Transcriptome changes occurring during ES differentiation or iPSCs generation revealed regulation in the expression of several RNA processing factors, including RBM47, Zcch4 beside the previously mentioned MBNL1/2, ESRP1 and RBFOX2 [160, 202, 206]. Notably, key transcription factors regulating pluripotency also control the expression of splicing factors with a role in stem cell biology. For instance, SRSF2, which enhances self-renewal of ESCs by promoting MBD2c splicing (Fig. 3) and OCT4 and NANOG expression, is in turn positively regulated by OCT4 [208]. The cross-regulation between SRSF2 and OCT4 suggests the existence of a positive feedback loop between splicing regulators and transcription factors that reinforces stemness features. Importantly, this regulatory loop involves both transcriptional and post-transcriptional regulatory mechanisms, as OCT4 not only binds the SRSF2 promoter, but it also negatively regulates the expression of miRNAs targeting its 3′UTR, such as miR-301b and miR-130b [208]. Moreover, OCT4 promotes the expression of miR-302 family members [208, 219], which specifically target the somatic-specific MBD2a isoform, but not the stem-specific MBD2c variant [208], thus further reinforcing a stemness positive feedback-loop. Additional evidence highlighting the existence of a crosstalk between transcriptional and splicing regulators in stem cell biology arises from a recent study showing that early epigenetic reprogramming occurring during iPSCs induction is functional to control the expression of several splicing regulators leading to activation of an AS program that is crucial for reprogramming [220]. The histone acetyltransferase GCN5 functions as an early mediator of the global epigenetic changes occurring during early phases of iPSCs induction. GCN5 cooperates with the reprogramming factor c-MYC in the regulation of its target genes, including several splicing regulators such as such U2AF1, TRA2B and SNRP70. Depletion of GCN5/c-MYC impacts on the AS program activated during the early phases of somatic cell reprogramming, particularly affecting genes involved in the regulation of cell adhesion and migration [220]. Notably, AS itself may also contribute to regulate the expression of splicing factors controlling stem cells proliferation and differentiation. For example, RBFOX2, which promotes ESC differentiation, directly regulates the steady-state expression levels of several other splicing regulators by AS-NMD mechanisms [124, 221].

Collectively, AS regulation represents an optimal tool to maintain stem cell plasticity and redefine developmental fate according to differentiation signals.

### Alternative splicing regulation in CSCs

Acquisition of stem-like features in more aggressive cancer cells has been frequently correlated to the expression of oncogenic splice-variants produced as a consequence of aberrant AS regulation. For instance, widespread alteration in the splicing programs of leukemia stem cells (LSCs) compared to normal stem and progenitor cells were revealed by high-throughput screenings in both chronic myeloid leukemia (CML) [222] and acute myeloid leukemia (AML) [223]. Both studies also identified a global dysregulation in the expression of genes encoding for spliceosomal proteins and RNA processing factors, further suggesting that aberrant AS regulation may contribute to LSCs generation [222, 223] and that this may occur independently from oncogenic mutations in splicing regulatory genes that are frequently observed in different types of leukemia [223, 224]. Interestingly, it has been recently suggested that downregulation of the splicing regulator MBNL3 in LSCs enhances splicing of the CD44 v3 isoform, which positively regulates their self-renewal capacity [225]. As previously described, MBNL3 belong to a family of splicing regulators that promote ESC differentiation [206]. Thus, aberrant splicing events observed in CSCs may be correlated to reactivation of embryonic splicing programs [225], similarly to what described for the activation of the EMT pathway [226]. This hypothesis is consistent with the expression of other oncogenic/embryonic AS variants in cancer cells. For instance, PKM2 is the embryonic splice-variant of the *PKM* gene that promotes aerobic glycolysis and sustains cancer cells proliferation and metabolism [227]. Interestingly, one of the four transcription factors necessary for iPSCs induction, c-MYC [228], induces the expression of oncogenic splicing factors (PTBP1 and hnRNP A1/A2) in cancer cells, which in turn promote PKM2 splicing [229]. Notably, promotion of PKM2 splicing was recently shown to confer chemotherapeutic resistance in pancreatic cancer [230]. Conversely, the tumor suppressor RBM4 [231] promotes neuronal differentiation of human mesenchymal stem cells by enhancing PKM1 splicing [232], thus further suggesting that modulation of the embryonic splicing program might regulate acquisition and maintenance of stemness features.

Splicing events supporting stemness and proliferation of CSCs have been described for genes involved in different cellular functions, such as apoptosis, signal transduction and cell-adhesion. For example, LSCs were shown to express high levels of the anti-apopoptic splice variants of the *BCL-2*, *MCL1*, *BCLXL*, and *BFL1* genes [233], as well as an AS variant of the *GSK3-β* gene that increases LSCs self-renewal [234]. High expression levels of the splicing regulator PTBP1 in brain tumor cells lead to skipping of exon 6 in the *ANXA7* transcripts, generating a shorter isoform of this membrane protein, named isoform 2, which enhances EGFR signaling and promotes cell tumorigenicity [235]. A common splicing event in CSCs of different tumor types is the inclusion of the variable exons of the *CD44* gene. Expression of the CD44v variants is displayed in both LSCs [225] and CSCs of solid tumors, such as colon [236] and gastric [237] cancers, with each tumor type expressing one or more specific variable exons: v3 in LSCs, v6 in colon cancer and v8-10 in gastric cancers. Moreover, splicing of the variable exons of v8-10 has been shown to promote CSC-like features in prostate cancer cells [238] and to increase the invasive and tumorigenic potential of bladder cancer cells [239]. Several splicing factors have been shown to enhance splicing of the CD44 variable exons in cancer, such as SAM68 [240], RBM3 [238] and ESRP1 [120], suggesting that regulation of their expression or activity may underlie CD44 splicing control in CSCs. Intriguingly, CD44v splice variants represent a marker of CSCs even though they are considered epithelial isoforms. Indeed, as aforementioned, the switch from a CD44v toward a CD44s splicing pattern under the control of ESRP1 has been correlated with the EMT of both mammary [154] and bronchial epithelial cells [153]. However, expression of epithelial markers by stem cells is not completely surprising, as a MET phase occurs also during reprogramming of somatic cells into iPSCs [241]. It is thus conceivable that expression of CD44v in CSCs is functional to the re-establishment of an epithelial phenotype, which allows engraftment of cancer cells in the site of secondary lesions during metastasis. Moreover, considering the high heterogeneity in CD44 isoforms expressed by CSCs, which has been documented in breast cancer [242], it is also plausible that regulation of *CD44* splicing may allow CSCs to maintain the hybrid E/M state that has been correlated with higher stemness and tumorigenicity [51, 243]. Regulation of *CD44* splicing clearly demonstrates the great impact that this post-transcriptional regulatory mechanism exerts on CSCs biology, paving the way for further studies aimed at identifying new splice variants and splicing regulators that may represent valuable targets for new approaches interfering with CSCs phenotypic plasticity.

## Conclusions

Epithelial and mesenchymal cells, as well as pluripotent and differentiated cells, represent extreme edges of tightly regulated processes: EMT and stem cell differentiation, respectively. In cancers, EMT is linked to metastasis formation as well as CSC generation and maintenance. Tumor populations are highly heterogeneous. Indeed, not all cancer cells are able to undergo EMT at the same time and not all cells that have activated an EMT program become competent to form metastasis. Tumor heterogeneity is further increased by the existence of epithelial/mesenchymal hybrids in highly metastatic CTCs and CSCs. Together these findings strongly suggest the importance of cellular plasticity for the acquisition of both invasive capabilities and stemness traits.

High-throughput approaches have recently documented remarkable changes in AS profiles of specific genes during activation of EMT programs and CSC generation. Frequently, such alterations are caused by changes in the expression levels of *trans*-acting factors. These analyses point out that AS provides an additional and extremely flexible layer of regulation to rapidly control temporal and spatial expression of protein isoforms, thus shaping cell- and tissue-identity. Importantly, AS variants orchestrate several important aspects of the EMT process, including cell-cell contacts, polarity and cytoskeleton organization, and CSC self-renewal and differentiation. Moreover, the pivotal role of AS regulation in tumor plasticity is underscored by the observation that this mechanism rapidly shifts the expression of protein isoforms with opposite functions. Finally, the recent optimization of antisense oligonucleotides-based approaches to selectively control splicing switches [244–246] suggests that AS variants specifically expressed during tumor EMT and in CSCs could represent valuable diagnostic or therapeutic options for anti-cancer purposes in the near future. However, although an enormous work in the field has already been done, the examples that we have discussed likely represent just the tip of the iceberg, and much more remains to be uncovered in order to draw a more realistic picture. Thus, future studies are warranted to fully elucidate the real contribution of AS regulation to cancer progression.

## Abbreviations

AJ: Adherens junctions
AML: Acute myeloid leukeia
AS: Alternative splicing
AS-NMD: Alternative splicing - non-sense mediate decay
CML: Chronic myeloid leukemia
CSCs: Cancer stem cells
CTCs: Circulating tumor cells
DS: Desmosomes
ECM: Extra-cellular matrix
EMT: Epithelial-to-mesenchymal transition
EMT-TF: EMT-transcription factor
ESCs: Embryonic stem cells
iPSCs: Induced Pluripotent Stem Cells
LSCs: Leukemia stem cells
MET: Mesenchymal-epithelial transition
MMPs: Metalloproteases
RBPs: RNA binding proteins
RNAi: RNA interference
TJ: Tight junctions
